# Supplementary material for: Identification of a Maturation Plasma Cell Index through a Highly Sensitive Droplet Digital PCR Assay Gene Expression Signature Validation in Newly Diagnosed Multiple Myeloma Patients
Source: Int J Mol Sci. 2022 Oct 18;23(20):12450. doi: 10.3390/ijms232012450 (PMC9604171; doi:10.3390/ijms232012450)
Supplement: Supplementary file 1 [file ijms-23-12450-s001.zip › ijms-1919686-supplementary.pdf]

SUPPLEMENTARY INFORMATION

1) **Multiple Myeloma CD138+ at diagnosis – GPL570 (Genechip U133 Plus 2.0)**

**Series**	**Accession: GSE87900**	**ID: 200087900**
**Series**	**Accession: GSE159289**	**ID: 200159289**
**Series**	**Accession: GSE82307**	**ID: 200082307**
**Series**	**Accession: GSE31161**	**ID: 200031161**
**Series**	**Accession: GSE19784**	**ID: 200019784**

2) **CD138+ cells from healthy donor - GPL570 (Genechip U133 Plus 2.0)**

**Series**	**Accession: GSE5900**	**ID: 200005900**
**Series**	**Accession: GSE36474**	**ID: 200036474**

Table S1. GEO Datasets downloaded from GEO databases (https://www.ncbi.nlm.nih.gov/gds)

HH GENES AFFY ID TARGET SEQUENCES

**SUFU**	SUFU - 222749_at
**SMO**	SMO - 218629_at
**SHH**	SHH - 207586_at
**PTCH2**	PTCH2 - 221292_at
**PTCH1**	PTCH1 - 209816_at
**IHH**	IHH - 229358_at
**GLI3**	GLI3 - 1569342_at
**GLI2**	GLI2 - 207034_s_at
**GLI1**	GLI1 - 206646_at
**DHH**	DHH - 1552730_at

SUFU

cagctgcaattctgagggggggtttgggaggcttgtgcgaggtctcaggcctgtgtggggagctggtgcctcttcctgcccgtatctttctcttccaagggcagtg
ctccaaggcagggactggagaagccaaggggagagtctaaaagggctagagcatttttaaaaatagacacagggtcttgggactggggtttcggattgagtt
gcaagcagggagaaaacctgaaggtcggtgcccctatggggctgaccagtagagaatttcctttactgtatttttgtgtctggtcttccctttctggcttctaggac
atccatgccaggtgaggtgcctgggtccctgttacaagtcaggagccctgtagggagacccctccttttgtacaagtacctgaatgctgcgacaagcagattttt
gtaaaattttatattagtttttaatgtcagtggcgactcggttcctggggctgcagccagcctgggacttttgtaagaatttttgggtgactcacttagatgtcgtttcc
ttcttgc

F: CTTCCCTTTCTGGCTTCT
R: GGCTCCTGACTTGTAACA

Probe Sequence: ACCTCACCTGGCATGGATGTCC
Flurophore: FAM

SMO

taacatctccatggggaggcctcaccccagggacagggccctggagctcagggtccttgtttctgccctgccagctgcagcctggttggcagcatctgctcca
tcggggcagggggtatgcagagcttgtggtggggcaggaacggtggaggcagaggtgacagttcccagagtgggctttggtggccagggaggcagccta
gcctatgtctggcagatgagggctggctgccgttttctgggctgatgggtgcccttcctggcagtctcagtccaaaagtgttgactgtgtcattagtcctttgtcta
agtagggccagggcaccgtattcctctcccaggtgtttgtggggctggaaggacctgctcccacaggggccatgtcctctcttaataggtggcactaccccaa
acccatcttttgttctcctatatcctccttctcctgttccatttcagttcagt
F: GCAGCCTAGCCTATGTCT
R: GACTGAGACTGCCAGGAA
Probe Sequence: ACGGCAGCCAGCCCTCATCT
Flurophore: FAM

SHH
gagcccgaggcgtcctcgggctcggggccgccttccgggggcgcactggggcctcgggcgctgttcgccagccgcgtgcgcccgggccagcgcgtgta
cgtggtggccgagcgtgacggggaccgccggctcctgcccgccgctgtgcacagcgtgaccctaagcgaggaggccgcgggcgcctacgcgccgctca
cggcccagggcaccattctcatcaaccgggtgctggcctcgtgctacgcggtcatcgaggagcacagctgggcgcaccgggccttcgcgcccttccgcctg
gcgcacgcgctcctggctgcactggcgcccgcgcgcacggaccgcggcggggacagcggcggcggggaccgcgggggcggcggcggcagagtag
ccctaaccgctccaggtgctgccgacgctccgggtgcgggggccaccgcgggcatccactggtactcgcagctgctctaccaaataggcacctggctcctg
gacagcgaggccctgcacccgctgggcatggcggtcaagtccagctgaagcc
F: GCAGAGTAGCCCTAACCG
R: TGCGAGTACCAGTGGATG
Probe Sequence: TCCAGGTGCTGCCGACGCTC reverse: GAGCGTCGGCAGCACCTGGA
Flurophore: FAM

PTCH2
gggcatcaagctgagtgccatccccgtggtgatccttgtggcctctgtaggcattggcgttgagttcacagtccacgtggctctgggcttcctgaccacccagg
gcagccggaacctgcgggccgcccatgcccttgagcacacatttgcccccgtgaccgatggggccatctccacattgctgggtctgctcatgcttgctgggttc
ccactttgacttcattgtaaggtacttctttgcggcgctgacagtgctcacgctcctgggcctcctccatggactcgtgctgctgcctgtgctgctgtccatcctgg
gcccgccgccagaggtgatacagatgtacaaggaaagcccagagat
cctgagtccaccagctccacagggaggcgggcttaggtggggggcatcctcctccctgcc ccagagctttgccagagtgactacctccatgac
F: GCCCTTGAGCACACATTT
R: CAGCAAGCATGAGCAGAC
Probe Sequence: ACCGATGGGGCCATCTCCACATTG reverse:
CAATGTGGAGATGGCCCCATCGGT
Flurophore: FAM

PTCH1
gtgttggagccatcagaaactgttgtttcatcttgctgaccttgtgacactcttctttttgctctatctgatggagagagttttaggttttcctcctttttgttttgtataaat
agtgggtataagcatagctctgttacatggatgtattacaaagtgggggctgagcttttagtgtgatcatcacccaaatantgtacattgtacccattaattaatttct
tatcacctgaccctatttttgttttctcataccaagaaacttcctaagttaaccatcaaaattagtccttctgtca
tctctcttagcatacttatttcccttatgtgatgccta
F: GAGCCATCAGAAACTGTTGT
R: GAAAACCTAAAACTCTCTCCATCA
Probe Sequence: TCATCTTGCTGACCTTGTGACACTCTT reverse:
AAGAGTGTCACAAGGTCAGCAAGATGA
Flurophore: FAM

IHH
cagcatggatctggctggacccgatgctgtccagaactgggaaggccacaggggtggggcagccatcccggccattntgaggtatgacattcctccccggc
cacactcctcaagacacatccagagactgttgctgtctgtgggcagagttctgtgttctggccaatgtgaccgtagtgccggggactgggggaggtgggttgg
atgtgcttgccaccccccggntaagctccccttntgntgaaccatgatccccaccccctccgccggtcagtctcccataccttatttattggag
tggagggggaagcccatgggagaattttg
F: CCACACTCCTCAAGACACAT
R: CGGCACTACGGTCACATT
Probe Sequence: ACACAGAACTCTGCCCACAGACAGCAA
Flurophore: FAM

GLI3
tacatccacctagagagtcagaatctcctttggaaggtaactgcagtccagaacattagactctaagggcatatacttccaagcagcctggccacttctctctgg
gtccctccatgtctgctgagagcctgtgcttctcacctgcacttcttcacccggtttcatgagtgtgctttgctgactgtactgggtagggaaggagctggctctta
aacctgctggcaaatgacttcagttttaaattgatcctgagggagatatatttacgtctgagttaaaataatgaatttacatatatgtgaaaatatttcctcctttattgat
ggtatttaaagtcaagactggagcttattttaattg agccagaatatttgcatttttcatccantnnnnttatagaaggcatttctccttagaaatatgttaccttgg
F: CCACCTAGAGAGTCAGAATCT
R: AGGCTGCTTGGAAGTATATG
Probe Sequence: ATGTTCTGGACTGCAGTTACCTTCCAA
Flurophore: FAM

GLI2
tgagtgcacccggaggggtcatcgctgcccagagcctggggattccagctgtcttgtcttttttccaaaaaagtgttaaataggcttgaggggttgttgcgcaatg
gccgcttcagatgacagatgttgtaagagaaggtttatgggcatcctctctggtcttttggattattcctcagaacaatgaaaaaagtctccataggacaggaagg
aatgcaaaactcatttacacagtgctttccagcctttggtgcttacaggaccgcgctgttccggcttcttcacggctgacattcggctaacgagggattactttggc
caaaacctttcaaaggatatgcagaaagatggtagggagcatttgggtttgaatctgaatgctatactggatactctgctccggaaagatgagctttttattctact
acttggaaggaaaaggaattcctctatgaagcctaactcttgaggtctctaa
F: CTTCTTCACGGCTGACATT
R: ATGCTCCCTACCATCTTTCT
Probe Sequence: TGGCCAAAGTAATCCCTCGTTAGCC
Flurophore: FAM

GLI1

gcttcacatagggcagcagcaccacctcgacttctgcccccattgcccacttgctatgggcctctcaaagtgggaggcacaaaccccagctgtggtcatcctg
aggtgggcaggctaggagggggtcctgccttgtaccctcctcccgaaggacaggtatgtaaccccctggactctcttgatcttgacaacactcagctggacttt
gtggctattctggatgagccccaggggctgagtcctcctccttcccatgatcagcggggcagctctggacataccccacctccctctgggccccccaacatgg
ctgtgggcaacatgagtgtcttactgagatccctacctggggaaacagaattcctcaactctagtgcctaaagagtagggaatctcatccatcacagatcgcatt
tcctaaggggtttctatccttccagaaaaattgggggagctgcagtcccctgcacaagatgccccagggatgggaggtatgggctgggggctatgta

F: AAAGTGGGAGGCACAAAC

R: GTTACATACCTGTCCTTCGG

Probe Sequence: CCTCCTAGCCTGCCCACCTCA

Flurophore: FAM

DHH

ttgctacgcggttctggagagtcaccagtgggcgcaccgcnnttttgccccccttgagactgctgcacgcgctaggggcgctgctccccggcggggccgtcc
agccgactggcatgcattggtactctcggctcctctaccgcttagcggaggagctactgggctgagcgtcccaggcatagaaacctcgaagcgcccgagga
aacgagggcctgctggctgagatatggggctgtgggcagcagacgatgccgactggaagggagggagagggagggggagggagaaaatggggctatg
cctggcttaggggcaacctcgtactgagaggaggtgataccgggtcctaggtaggtagggctgatggtgcgcactccttaaagaggactatttagcctctccc
ccagtgcttcagccccacccgataattttattttatttcttatttataaattgtaatataatgattcccttgcccagcttgccaaggtgaggggctggggcatggcatta
acactgtctgacacagccagccantctgacatctgacgttttcctgcaaggtgggct

F: GACGATGCCGACTGGAAG

R: ATCACCTCCTCTCAGTACGA

Probe Sequence: AGGCATAGCCCCATTTTCTCCCTCCC

Flurophore: FAM

Table S2. Digital PCR assays designed for the 10 HH-genes signature validation. In order to design a 10-HH gene signature ddPCR assays, the exact probe sequences of the 10 HH genes were extrapolate (~500 bp each) from the Plus 2.0 gene chip array (*see Supplementary*). Custom ddPCR assays consist of two primers, forward and reverse, and an intermediate probe conjugated with fluorophore FAM and HEX, to possibly multiplex all the assays in a unique cartridge.

Sample	CLU1(D)	CLU2(D)	Classification
MM_01	3,17759	4,55191	1
MM_114	1,72194	3,85961	1
MM_125	1,4008	2,98937	1
MM_130	1,04041	3,36722	1
MM_132	2,77853	5,3267	1
MM_135	2,80261	4,56471	1
MM_137	1,55787	2,8444	1
MM_142	2,07608	5,50564	1
MM_144	1,71192	2,50349	1
MM_147	1,54728	4,63888	1
MM_155	1,44952	4,06789	1
MM_157	2,44405	4,64537	1
MM_159	2,10535	4,49955	1
MM_164	1,98867	2,78614	1
MM_31	1,26453	3,1201	1
MM_43	1,2523	2,70873	1
MM_48	2,65599	3,0948	1
MM_50	2,18393	2,90514	1
MM_56	1,87245	2,80158	1
MM_57	2,11503	4,43288	1
MM_85	0,92935	3,48978	1
MM_86	1,70745	3,32771	1
MM_95	1,59981	2,80787	1
MM_97	2,68989	4,65419	1
MM9n	2,78342	4,24884	1
MM16n	1,94185	4,06244	1
MM18n	2,37234	4,2569	1
MM19n	2,81017	4,39028	1
MM21n	1,99441	2,81729	1
MM22n	2,86907	4,52747	1
MM23n	2,57734	3,44307	1
MM_06	4,7177	1,81938	2
MM_07	4,61216	2,18063	2
MM_105	3,69741	2,54024	2
MM_108	3,40427	1,66657	2
MM_19	3,77439	1,52507	2
MM_20	4,55705	2,1031	2
MM_22	5,16039	2,70444	2
MM_32	3,24364	0,97954	2
MM_38	2,71083	1,54766	2
MM_40	3,44542	1,4175	2
MM_42	3,94145	1,19874	2
MM_55	3,66968	1,9453	2
MM_72	4,56268	1,94027	2
MM_88	4,67004	2,7424	2
MM_93	2,54525	0,98873	2
MM_96	3,0513	1,5642	2
MM_99	3,54276	1,02249	2
MM17n	3,47831	2,04719	2
MM20n	3,72712	2,37094	2

Table S3. Classification of 50 patients' test set according to the 10 HH-genes *signature* ddPCR assay. 31 patients were assigned to Cluster 1 and 19 to Cluster 2, according to the distance (D) of gene expression values from both clusters. The comparison of gene distances obtained both from gene expression and ddPCR data demonstrated a good correlation ($p = 0.0001$; $r^2 = 0.98$).
